# Pathological Changes and Sodium Rhodizonate Test as Tools for Investigating Gunshot Wounds in Veterinary Forensic Pathology

**DOI:** 10.3390/ani14192913

**Published:** 2024-10-09

**Authors:** Giuseppe Piegari, Ilaria d’Aquino, Giovanni Valerio Salanti, Vittoria Romano, Gianluca Miletti, Emanuela Sannino, Evaristo Di Napoli, Lorenzo Riccio, Davide De Biase, Orlando Paciello

**Affiliations:** 1 Department of Veterinary Medicine and Animal Production, University of Naples “Federico II”, 80137 Napoli, Italy; valeriosalanti@yahoo.it (G.V.S.); evaristo.dinapoli@unina.it (E.D.N.); lorenzo.riccio@unina.it (L.R.); paciello@unina.it (O.P.); 2 Istituto Zooprofilattico Sperimentale del Mezzogiorno, 80055 Portici, Italy; gianluca.miletti@izsmportici.it (G.M.); emanuela.sannino@izsmportici.it (E.S.); 3 Department of Pharmacy, University of Salerno, 84084 Fisciano, Italy; ddebiase@unisa.it

**Keywords:** forensic science, gunshot residues, penetrating injuries, veterinary forensic pathology

## Abstract

**Simple Summary:**

Gunshot residues (GSR) are particles produced during the discharge of a firearm. The typical composition of GSR is lead, barium, and antimony. The aims of our study were to (1) investigate the gunshot wound morphology and lead residues in animals and (2) correlate the morphology of the gunshot wounds and the distribution of lead residues with the range of fire. To these aims, cadavers with antemortem and experimentally produced post-mortem gunshot wounds were investigated. Morphological analysis was performed on all entry gunshot wounds. Lead residues were evaluated using macroscopic and histological colorimetric tests. Animals that died of firearm-related injuries showed wounds characterized by an abrasion ring associated with signs of tissue vitality. Experimentally shot limbs showed injuries related to the shooting range. Lead was observed on both animals that died from gunshot wounds and experimentally shot limbs. However, the number of positive cases and the pattern of lead distribution varied with the range of fire. Our results suggest that both pathological examination and colorimetric tests represent valid tools for investigating gunshot wounds in veterinary forensic pathology.

**Abstract:**

Gunshot wound morphology and gunshot residues (GSRs) evaluation have been poorly investigated in veterinary forensic pathology. The aims of the present study were to assess the gunshot wound morphology in animals and evaluate the detectability of lead deriving from GSRs using colorimetric techniques. To these aims, cadavers were divided into four different groups. Group A comprised eight animals who died from firearm-related injuries, while groups B and C included dog limbs shot using different shooting ranges; group D comprised dog limbs stabbed with a screwdriver. Morphological analysis was performed on all entry gunshot wounds. Lead residues were investigated using a *Bullet Hole Testing Kit* (*BTK*) *and Rhodizonate Sodium histochemical staining* (*NaR-s*). Gunshot wounds in group A showed an abrasion ring associated with hemorrhages and tissue necrosis. Groups B and C showed injuries related to the shooting range. NaR-s showed positive results in both animals that died from gunshot wounds and experimentally shot limbs. However, the number of positive cases and the pattern of lead distribution varied with the shooting range. Positive results by BTK were limited to close-contact shots in group B limbs. Our results suggest that both pathological examination and NaR tests represent valid tools for investigating gunshot wounds in veterinary pathology.

## 1. Introduction

Penetrating injuries can be defined as “*any injury caused by a foreign object piercing the skin, which damages the underlying tissues and results in an open wound*” [1]. These can be classified into two different groups: gunshot and sharp force traumas [2]. Gunshot wounds constitute a class of penetrating injuries caused by a projectile ejected from firearms following the ignition of gunpowder [3]. By contrast, sharp force injuries are lesions caused by objects not related to firearms, such as knives or ice picks [4]. In forensic practice, the observation of single or multiple entry wounds is necessary to define an injury as penetrating; furthermore, the detection of both entry and exit wounds allows for the definition of a penetrating trauma subtype called perforating trauma [5,6]. A penetrating injury associated with the detection of a bullet inside a victim’s body is usually sufficient to make a diagnosis of gunshot trauma; however, perforating wounds require a more in-depth forensic investigation in order to correctly define the etiology of the trauma. In these cases, a complete evaluation of the crime scene, post-mortem macroscopic and histological findings, and ancillary examinations are necessary before making a final diagnosis. Overall, gunshot wound morphology is strongly influenced by different variables, such as the weapon’s caliber and shooting range, with the latter usually classified as contact, near-contact, intermediate, and long-range based on the range of fire [7]. In human forensic pathology, both contact- and intermediate-range wounds are characterized by distinctive traits that can allow for easier injury identification [7,8]. These features are a consequence of the soot deposition on the skin (soot deposition around the entry wound and soot cavity), the searing of skin surrounding the entrance wound (muzzle flash), and the embedding of soot particles in the skin (powder tattooing) [7,8]. Unfortunately, the morphology of the lesion is much simpler and more misleading in distance-range wounds; in these cases, the gunshot wound is only characterized by skin lacerations and an abrasion ring caused by the projectile [7,8]. To overcome this limit, many techniques have been validated in human forensic pathology to detect the presence of gunshot residue on surfaces, textiles, or gunshot wounds in cadavers. Gunshot residues (GSRs) can be generally defined as “*the different particles expelled during the discharge of a firearm*” [9] and are mainly composed of lead, barium, and antimony, which are derived from the primer cap and from gunpowder; their detection and pattern of distribution can be used to both confirm gunshot trauma and estimate the range of fire [9,10,11]. Currently, the methods applied in the detection of GSRs comprise *colorimetric tests, instrumentation-based analysis, and electrochemical techniques* [12,13,14]. Among them, the sodium rhodizonate (NaR) test is a colorimetric test based on a chemical reaction between Na-rhodizonate and lead residues. The NaR was initially employed as a macroscopical test to detect the presence of lead on a victim’s clothes and on the shooter [15]; subsequently, it has been applied as staining for the histological analysis of a victim’s gunshot wounds [16]. Unfortunately, although the gross and histological characteristics of gunshot wounds have been extensively investigated in humans [7,8,17,18,19], few studies have evaluated their morphology in animals [20,21,22]. Furthermore, no reports have investigated the macroscopic application of the NaR technique in veterinary forensic pathology. In addition, the histological validation of NaR staining is currently limited to human and animal models, such as pigs [16,18,21,23,24]. Finally, no studies have evaluated the histological patterns of lead deposition according to firing distances in dogs. Although studies conducted on humans have provided valuable information in veterinary forensic sciences, the morphological differences between species do not allow for the direct application of assessed parameters to animals. Similarly, the peculiar anatomical characteristic of pigs (the absence of fur and skin pigmentation) makes it difficult to apply the acquired information to other animals of veterinary forensic interest. In light of these observations, the aims of this study were to (1) investigate the pathological findings of entrance gunshot wounds in animals that died as a result of firearm-related injuries and in dog’s cadaver limbs shot at different shooting distances; (2) evaluate the detectability of lead derived from GSRs, using both macroscopic and histological colorimetric techniques; (3) correlate the pathological findings and the patterns of NaR chromogenic reactions with range of fire; (4) introduce a standardized forensic protocol for the diagnostic investigation of gunshot wounds in veterinary forensic practice.

## 2. Material and Methods

### 2.1. Study Design

A total of fifteen dead animals were enrolled in this study and divided into four groups. Group A included eight animals (three cattle, three dogs, two horses) that died from gunshot wounds, whereas groups B, C, and D included eighteen, six, and four dogs’ limbs, respectively. The limbs were obtained from seven dogs that died from different causes unrelated to trauma and in which a prior gross examination excluded infectious disease. The samples were transported, in sealed containers, to the shooting range, “Tiro a Segno Nazionale Napoli”. The limbs from group B were experimentally shot with a pistol (*model Glock 17*) using both 9 mm copper-plated bullets and 9 mm full metal jacket bullets at close contact (0 cm), intermediate (1 m), and long (6 m) distances. The limbs from group C were experimentally shot using a rifle with a caliber 12 shotgun slug. Considering the higher firepower of the rifle compared to the pistol, the limbs in group C were shot at 6 and 12 m distances. Finally, the samples from group D (the control group) were stabbed with a screwdriver to mimic gunshot trauma. The experimentally induced shot or stabbing was performed on the lateral-proximal portion of the limbs. Table 1 summarizes the manner of death of the animals that died from gunshot wounds (group A); Table 2 summarizes the kind of firearm, bullet, shooting range, and number of samples used for the experimental section of the study (groups B and C).

### 2.2. Gross Examination and Macroscopic Chromogenic Test

Post-mortem examinations and macroscopic evaluations of lead residues were performed in all the assessed cases. A forensic gross examination was performed in the necropsy room at the Department of Veterinary Medicine and Animal Production of the University of Naples “Federico II” using a previously described protocol [25]. The macroscopic evaluations of lead residues were conducted using the *Bullet Hole Testing Kit* (*BTK*). The BTK was applied according to the user manual as follows: lead solvent was placed on the surface of a test paper, which was then pressed onto the gunshot wounds for 30 s; subsequently, lead reagent was applied on the test samples; positivity, if present, was shown as a red color, while yellow represented a negative reaction [26].

### 2.3. Histological and Histochemical Examination 

At the end of the macroscopic evaluation, representative samples of entrance gunshot wounds were collected for histopathologic analysis. The samples were fixed in 10% neutral-buffered formalin, embedded in paraffin, sectioned into 4 microns, and stained with hematoxylin and eosin (HE) for subsequent morphological wound evaluations [27]. Sodium rhodizonate histochemical staining (NaR-s) and its variant with hydrochloric acid (NaR HCL 5%) were used to investigate the lead residues. NaR-s and NaR HCL 5% were performed according to protocols previously described [28]. The pattern of distribution of Rhodizonate Sodium positive deposits was classified in the continuum, scattered and dotted. Sections were evaluated by two independent pathologists (G.P. and D.D.B.).

## 3. Results

### 3.1. Gross Examination

**Figure 1:**
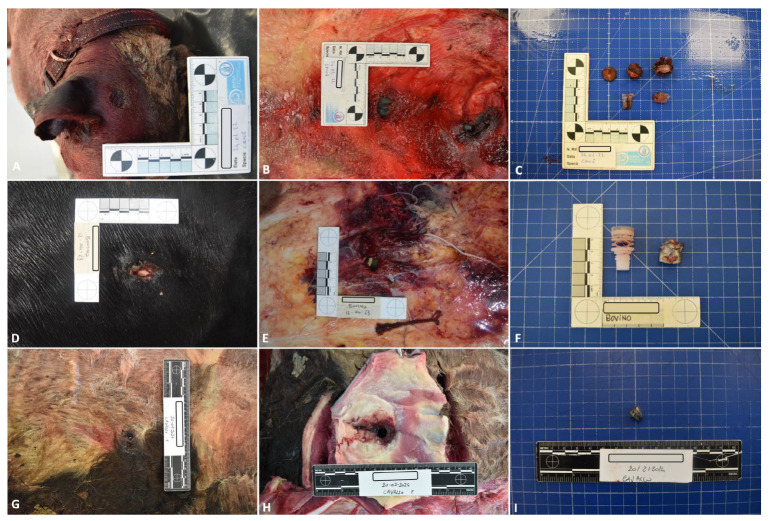
Representative pathological and ballistic findings in group A cadavers; dog: (**A**) gunshot wound (**B**) subcutaneous hemorrhages around the entrance gunshot wound (**C**) ballistic material detected during the necropsy; bovine: (**D**) gunshot wound (**E**) subcutaneous hemorrhages around the entrance gunshot wound (**F**) ballistic material detected during the necropsy; horse: (**G**) gunshot wound (**H**) subcutaneous hemorrhages around the entrance gunshot wound (**I**) ballistic material detected during the necropsy.

Group A—The entry gunshot wounds in group A were characterized by a circular or oval morphology with irregular and hemorrhagic edges; no soot deposits on their surface were observed in any of the assessed cases. Subcutaneous hemorrhages around the entrance gunshot wound and ballistic material in the subcutaneous tissue or internal organs were observed in all the assessed cases; gross examinations also showed intracavitary hemorrhagic effusion, bone fractures (dog: three out of three cases; cattle: two out of three cases; horse: two out of two cases), and internal organ lacerations (Figure 1). Table 3 summarizes the location of the gunshot wounds observed in the study animals.

**Figure 2:**
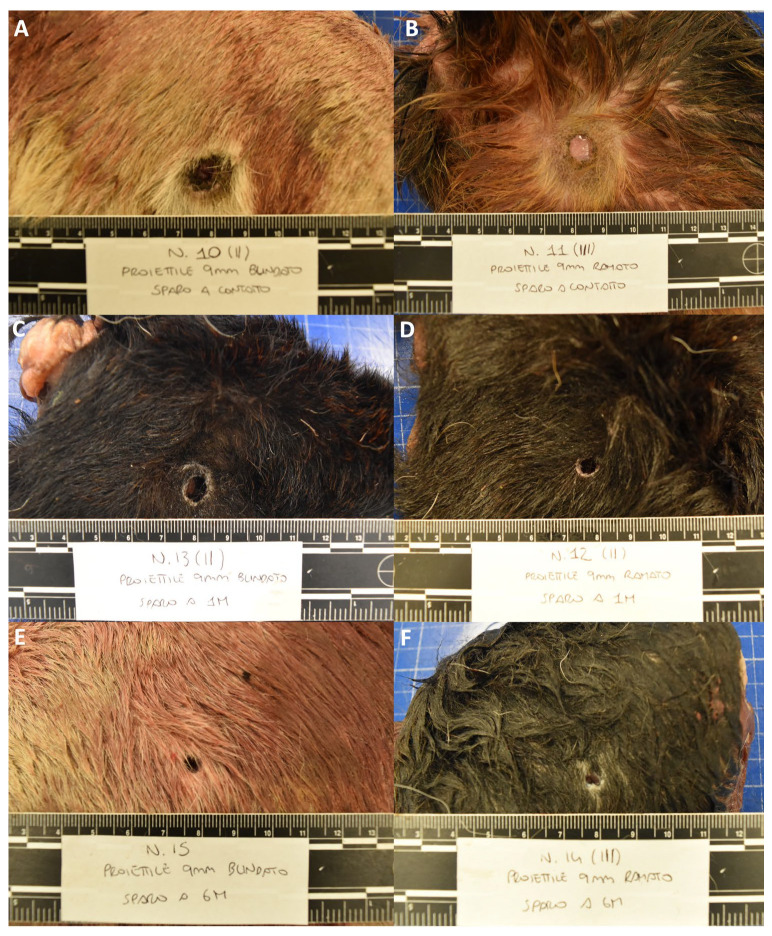
Representative morphological alterations in group B limbs; close contact shot: (**A**) full metal jacket (**B**) copper-plated; 1 m shot: (**C**) full metal jacket (**D**) copper-plated; 6 m shot: (**E**) full metal jacket (**F**) copper-plated.

**Figure 3:**
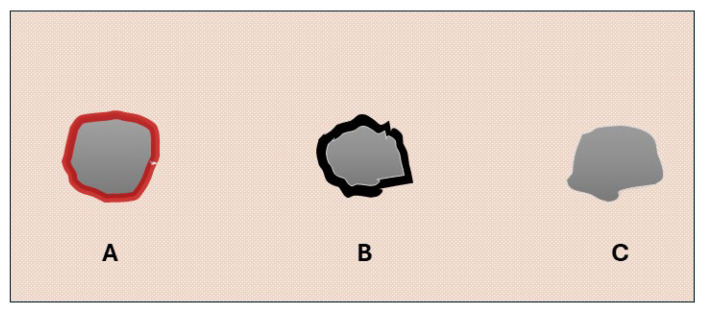
Graphical representation. (**A**): group A gunshot wound showing a circular morphology with irregular and hemorrhagic edges; (**B**): close contact shot in group B limbs showing an abrasion ring associated with a moderate amount of visible black material on the surface of the skin; (**C**): 1 and 6 m shots in group B limbs showing an abrasion ring without macroscopically visible signs of soot deposits.

Group B—The entry gunshot wounds in group B, which were caused by both copper-plated and full metal jacket bullets, showed similar morphologies. For close-contact shots, the gunshot wounds showed a round-to-oval shape, and their sizes ranged between 0.4 and 1.9 cm. The edges of the wound were irregular and appeared indented; along the margins of the wound, the skin appeared abraded and alopecic (abrasion ring), and differing amounts of visible black material (soot deposits) on the surface of the skin wounds were observed in all the assessed cases. The area surrounding the entry wound appeared seared, and, especially closer to the center, the fur was burned (muzzle flash). At a 1 m distance, the lesions showed an oval morphology, with sizes ranging between 0.4 and 1 cm. Although the skin appeared to be abraded (abrasion ring), no observable soot deposits or alterations due to the release of high-temperature gasses (muzzle flash) were observed in any of the assessed cases. Lastly, wounds from gunshots fired at a 6 m distance showed a rounder morphology, with margins that appeared less irregular. The sizes of these ranged between 0.4 and 0.6 cm. Similar to the 1 m range lesions, an abrasion ring was observed in all the assessed cases; however, no soot deposits or thermal changes were detected in any of them (Figure 2, Figure 3 and Figures S1–S15).

**Figure 4:**
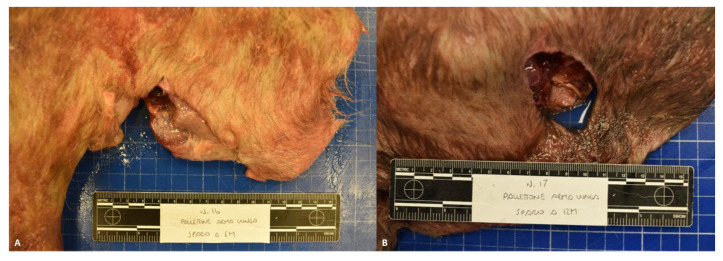
Representative morphological alterations in group C limbs; 6 m (**A**) and 12 m shots (**B**).

Group C—The wounds caused by a rifle shot fired at distances of both 6 and 12 m showed an oval shape with irregular edges and similar sizes (between 2 cm × 3 cm and 3 cm × 4 cm). No soot deposits were observed in any of the evaluated cases (Figure 4).

**Figure 5:**
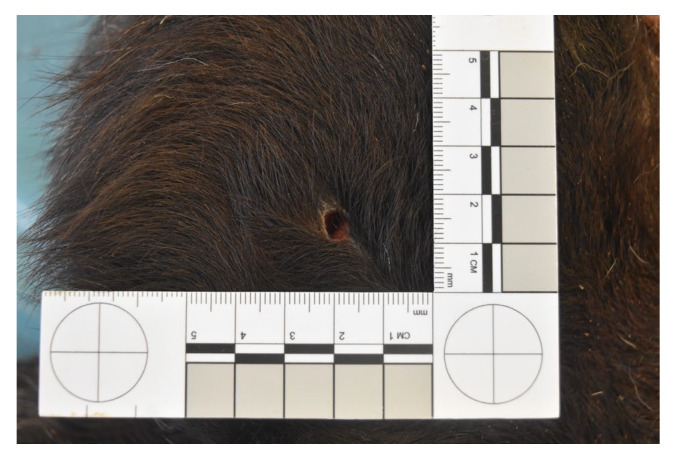
Representative morphological alterations in group D limbs.

Group D—The wounds caused by the screwdriver showed circular or oval shapes, with irregular margins and sizes ranging between 0.5 cm and 0.8 cm. No soot deposits were observed in any of the assessed cases (Figure 5).

### 3.2. Macroscopic Colorimetric Test (Bullet Hole Testing Kit)

**Figure 6:**
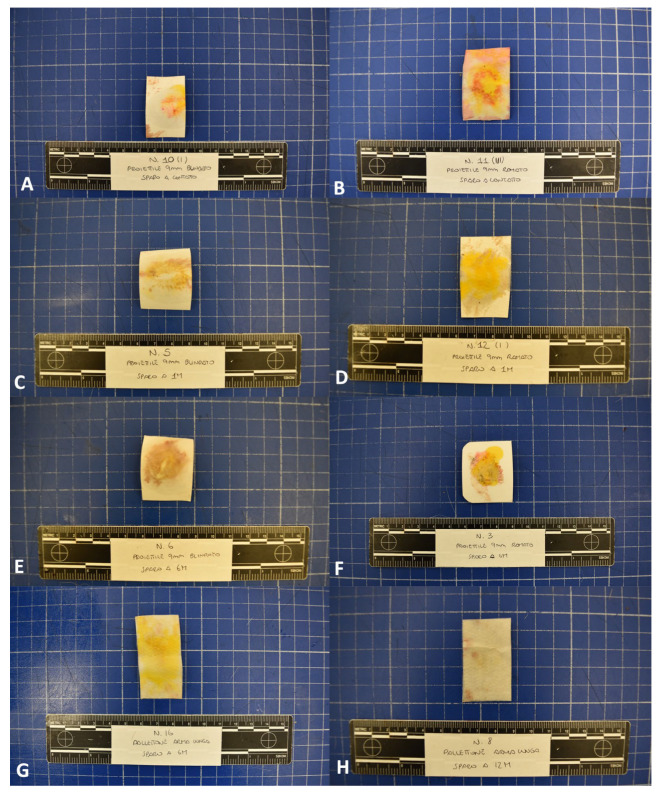
Bullet hole testing kit; *Pistol*: close contact shot (**A**,**B**) showing a pattern of lead distribution mimicking the shape of the entry wound in both (**A**) full metal jacket and (**B**) copper-plated cases; 1 m shot (**C**,**D**) and 6 m shot (**E**,**F**) showing negative results. *Rifle*: 6 m (**G**) and 12 m (**H**) shots showing negative results.

The macroscopic colorimetric test showed negative results in all cases in group A (*animal dead as a result of gunshot trauma*), group C (*limbs experimentally shot using a rifle at 6 and 12 m distances*), and group D (*control group*). For gunshot wounds in group B (*limbs experimentally shot using a pistol at 0, 1, and 6 m distances*), the macroscopic chromogenic test showed positive results in all close-contact cases (6/6; 100% of cases). In these cases, the pattern produced by the chromogenic reaction on the testing paper mimicked the shape of the entry wounds (Figure 6).

### 3.3. Histological and Histochemical Analysis (Sodium Rhodizonate Histochemical Staining)

**Figure 7:**
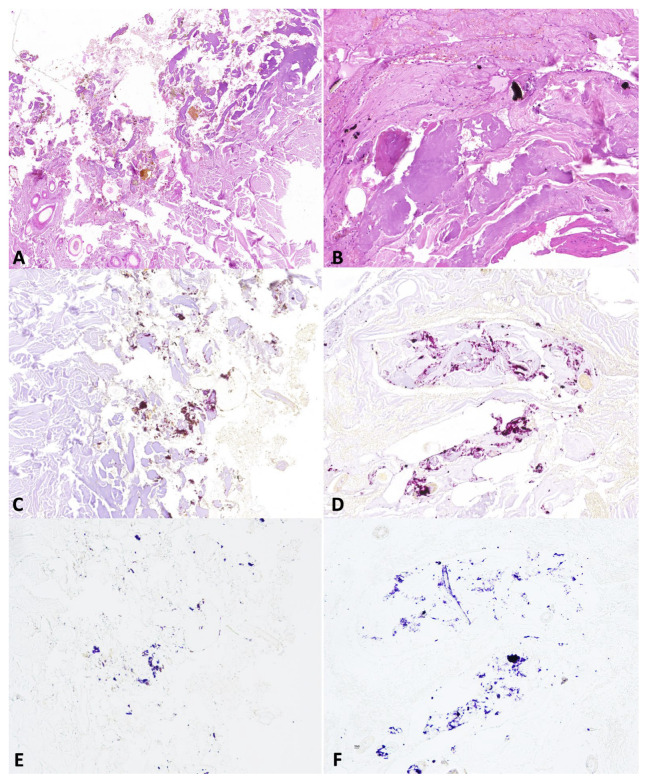
Representative skin morphological alterations in group A cadavers: (**A**,**B**) coagulative necrosis, hemorrhages and foreign material around the edges of the dog (A) and bovine (**B**) gunshot wounds (*Hematoxylin and eosin Stain, original magnification 20×*); (**C**,**D**) amorphous aggregates stained pink–brown with sodium rhodizonate in both dog (**C**) and bovine (**D**) gunshot wounds (*NaR, original magnification 20%*). (**E**,**F**) amorphous aggregates stained blue-violet with hydrochloric acid in dog (**E**) and bovine (**F**) gunshot wounds (*NaR HCL 5%, original magnification 20%*).

Group A—Histopathological analysis allowed us to detect multifocal or diffuse dermal necrosis and hemorrhages in all the evaluated cases. Moderate-to-severe neutrophil infiltration was observed in three out of eight cases. Hair fragments were also observed in the deep dermis or subcutis in five out of eight cases. Black deposits around the wound’s edges were detected in all evaluated cases. Staining with NaR and NaR HCL 5% showed multifocal positive aggregates on the surface of the gunshot wound in six out of eight cases (Figure 7) (dog: three out of three cases; horse: zero out of two cases; cattle: three out of three cases).

**Figure 8:**
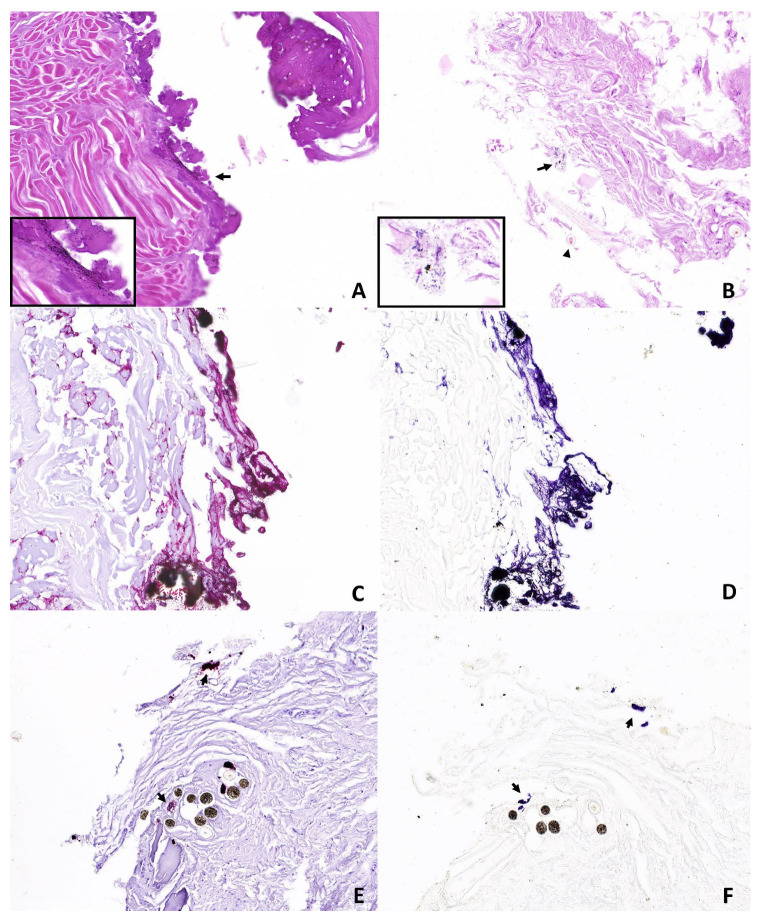
Representative skin morphological alterations in group B limbs: (**A**) close contact shot showing extensive protein agglutination and a moderate number of black deposits around the edges of the wound (arrow and insert); (**B**) intermediate shot range showing fragments of hair in deep dermis (arrowhead) and black deposits (arrow and insert) (*Hematoxylin and eosin Stain, original magnification 30×*). (**C**,**D**) close contact shot showing sodium rhodizonate positive deposits strongly clustered along the edges of the wound; (**E**,**F**) intermediate-range shot showing dotted positive aggregates (arrows) (*NaR-s and NaR HCL 5%, original magnification 30×*).

**Figure 9:**
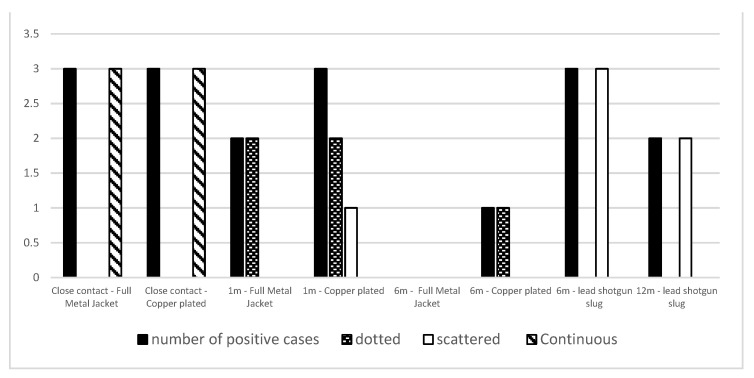
Number of positive cases and pattern of lead distribution for each assessed firearm (pistol and rifle), bullet (copper-plated, full metal jacket, and shotgun slug), and shooting ranges (close contact; intermediate and long-range).

Group B—Histopathological analysis allowed us to detect hair fragments in the deep dermis and subcutis. Extensive heat-induced protein agglutination associated with black deposits around the wound’s edges was observed in all close-contact shots. Variable amounts of black grain were also detected in intermediate- and long-range shots. Histochemical staining with sodium rhodizonate showed positive results in all close-contact shots. From shots fired at a 1 m distance, positivity was observed in three out of three cases for the copper-plated bullets and in two out of three cases for the full metal jacket bullets. For shots fired at 6 m distances, positive results were observed in only one case shot with copper-plated bullets. Furthermore, the pattern of sodium rhodizonate positivity varied among the assessed shooting ranges. Indeed, close-contact shots showed a continuous line of positive deposits strongly clustered along the edges of the injury. By contrast, a higher shooting distance showed a lower density of positive material localized focally or multifocally on the wound’s surface (Figure 8 and Figure 9).

**Figure 10:**
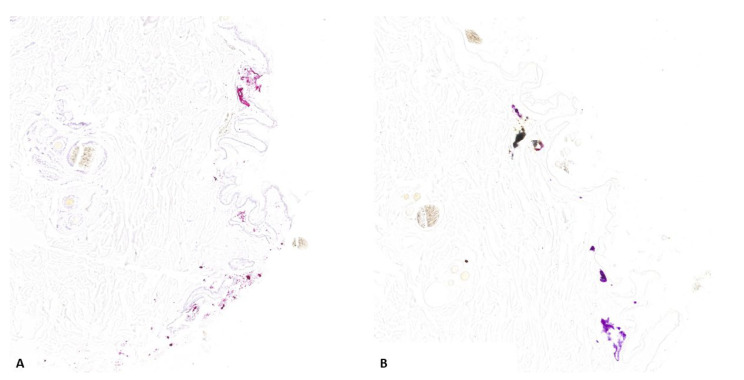
NaR-s and NaR HCL 5% in group C limbs: (**A**,**B**): long-range shot showing scattered positive aggregates (*NaR-s and NaR HCL 5%, original magnification 20×*).

Group C—Histopathological analysis allowed us to detect skin lacerations associated with hair fragments in the deep dermis and subcutis. Black deposits around the edges of the wound were observed for all the evaluated ranges of fire. Histochemical staining with sodium rhodizonate showed positive results in all cases shot at 6 m distances and in two out of three cases shot at 12 m distances; the distribution pattern was focally or multifocally scattered around the edges of the wound in all two of the assessed ranges of fire (Figure 9 and Figure 10).

**Figure 11:**
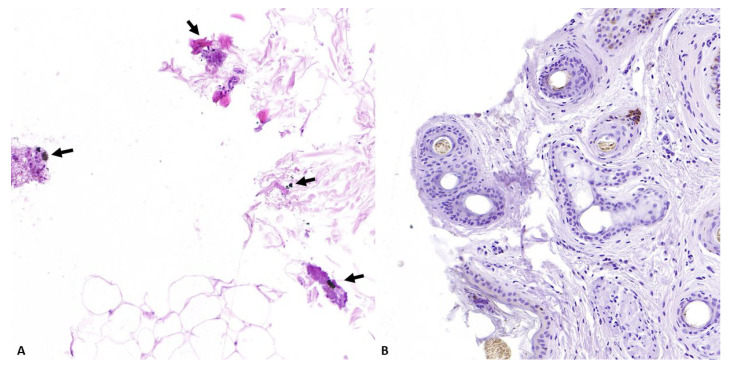
Representative skin morphological alterations in group D limbs: (**A**) moderate number of black deposits around the edges of the wound (arrows); absence of positive deposits (**B**) (*original magnification 30×*).

Group D—Histopathological analysis allowed us to detect skin lacerations without signs of thermal damage. Black deposits around the wound’s edges were observed in one out of four cases (Figure 11A), and histochemical staining with sodium rhodizonate did not reveal pink–brown or blue amorphous aggregates in any of the assessed cases (Figure 11B).

## 4. Discussion

Penetrating injuries can be a consequence of gunshot or sharp force traumas [1,2]. In these cases, a correct forensic approach is essential to define the etiology of the trauma and, in gunshot cases, identify evidence of gunshot residues on the surface of the victim’s body. Although gunshot injuries have been extensively investigated in humans and pigs [7,8,21], their morphological characteristics in other species are poorly examined. Moreover, studies on the application of colorimetric tests in the analysis of gunshot residues are confined to experimental work conducted on pigs’ cadavers and case-based studies on humans [18,21,24]. Consequently, significant limitations can be encountered as concerns about their application in veterinary forensic practice.

### 4.1. Macroscopic and Histological Findings

In our study, the entrance gunshot wounds both in animals that died from gun-related trauma (group A) and in experimentally shot limbs (groups B and C) showed some similar characteristics, such as a *round-to-oval shape, irregular edges, and surrounding abrasion collars*. These features are a consequence of skin laceration and over-stretching due to bullet penetration [7]. In addition to these findings, the cadavers in group A also showed gross and histological signs of tissue vitality, including hemorrhages and inflammatory infiltrate. These injuries confirm the ante-mortem nature of trauma and reflect the changes that occur immediately after tissue wounding in living vertebrates [29,30]. Nevertheless, the animals in group A showed no further macroscopic signs characteristic of gun-related trauma, such as soot deposits [7]. This possibility has been reported previously in human forensic pathology [23,31,32]. Indeed, soot deposits on the surface of a gunshot wound are a consequence of the different particles emitted during the shot [31]. Usually, the amount of soot on the surface of a gunshot wound is strongly influenced by different variables, such as the type of ammunition and range of fire [31,32]. Consequently, long-range fire or shot with specific ammunition can determine a lower amount or absence of this sign on gunshot wounds surface. Similar results were also observed in the experimental section of our study. In our case, macroscopically detectable soot deposits decreased with the increase in shooting range distance. Indeed, while differing amounts of macroscopically visible black material were observed on the wound surfaces in all close-contact cases in group B, no soot deposits were observed in wounds caused by intermediate- and long-range shots in both experimentally shot groups (*group B: pistol; group C: rifle*). Taken together, these findings are consistent with those reported by Zain et al. [32], who detected only a small amount of soot particles in white cotton fabric shot at a 50 cm distance using four types of firearms. However, our results are only partially in agreement with those reported by Hlavaty et al. [31], who reported macroscopically visible soot deposits, even at a 2.7 m distance, on pig limbs and heads shot using a 0.22 Long Rifle (22LR) and a 0.45 Automatic Colt Pistol. The differences between the aforementioned study and our results could be a consequence of both (1) the different ammunition and firearms used for the experiment and (2) the different anatomical structure of dog skin compared to that of pigs, mainly because of the presence of hair and skin pigmentation. Indeed, hair can act as a filter for GSR particles, resulting in lower soot deposition on the surface of an injury [33]. Furthermore, skin pigmentation may make soot on the surface of a gunshot wound more difficult to detect than in unpigmented skin. Associated with extensive soot deposits, the close-contact shots in group B showed signs of thermal damage surrounding the entrance wound. This morphological change has been observed both macroscopically and histologically and appears to be a consequence of the incandescent gasses released during the shot [7]. Furthermore, the histopathological examinations allowed us to detect black deposits around the edges of the wound in both experimentally shot and stabbed groups (groups B, C, and D) and animals that died from gunshot wounds (group A). As previously reported in the literature, these structures reflect the presence of gunpowder, lubricants, or other metallic structures from the bullet [18,19,31]. However, the detection of similar deposits in group D cases (control group) suggests a poor utility of their assessment in the investigation of gunshot wounds in dogs. Indeed, formalin, melanocytic, and hemosiderin pigments, as well as the dirt on the surface of the wound, can result in similar histological morphology [18,19]. Consequently, caution should be exercised in their evaluation of veterinary forensic pathology.

### 4.2. Macroscopic and Microscopic Colorimetric Tests

Lead is usually considered a characteristic inorganic component of gunshot residue. It is released from the primer, which is usually composed of antimony, barium, lead styphnate, lead dioxide, calcium, silicon, and tin [34,35]. Therefore, lead detection in wounds is strongly indicative of ballistic trauma in human forensic practice. In our study, BTK only showed positive results in the close-contact shots in the experimentally shot groups but in none of the animals that died from firearm-related injuries. These results appear to be different from those observed histologically, where positive results were observed in a greater number of cases, both in the experimentally shot cases and in animals that died from ballistic trauma. These findings suggest that the macroscopic chromogenic test has a lower level of sensitivity compared to the microscopic one. These data could be explained by (1) the interference of hair and organic material during contact between lead and the filter paper and (2) the low capacity of filter papers to collect a high amount of lead residue on the surface of a skin wound. As regards histochemical staining conducted on the cadavers in group A, positive aggregates were shown in six out of eight cases. The negative results observed in the remaining two cases could be a consequence of several factors, such as lead degradation due to decomposition processes [21], lead dilution due to environmental factors [36], or a lack of reactive material due to long-range shots [23]. Overall, the possibility of negative results in long-range shots was observed in the experimental section of our study. Indeed, a negative correlation between the range of fire and the number of positive cases was observed in group B. Specifically, histochemical staining with sodium rhodizonate allowed us to detect positive results in all close-contact shots in group B, where positivity at 1 and 6 meters in distance was observed in a lower number of cases. Furthermore, differences in patterns and the amount of lead distribution were found between close-contact shots and other shooting ranges. Taken together, these data reflect the progressive reduction in lead residues with increasing shooting distance [16,23]. Surprisingly, despite the larger shooting distances of the rifle compared to the pistol, group C showed positive results in almost all the evaluated cases, even at a 12 m distance. These results could result from the higher release of GSRs from the rifle ammunition compared to pistol ammunition [37]. Lastly, the negative results observed in the control group suggest a low selectivity of sodium rhodizonate staining for the structures of the normal dog skin and a low interference of environmental lead pollution on the results of the technique. These results are in apparent agreement with those reported by Borracchi et al. [38], which showed the absence of environmental lead contamination in both human cadavers found in open spaces and exhumed after a long period of time. Furthermore, our results are consistent with those reported by Andreola et al. [28], who reported a high selectivity of the NaR HCL staining for histological detection of lead. Indeed, sodium rhodizonate is a chromogenic reagent that can react with elements other than lead, such as barium, strontium salts, and hair follicles [8]. Nevertheless, the application of HCL can be used to confirm the reaction and improve the results in human forensic practice [28]. Taken together, our results suggest that the joint evaluation of both NaR and its confirmation with hydrochloric acid appears to be a useful screening tool to discriminate between gunshot and sharp penetrating traumas in veterinary forensic pathology.

## 5. Conclusions

The investigation of gunshot wounds is challenging in human forensic pathology as well as in veterinary forensic pathology. Our findings suggest that both pathological examination and colorimetric tests can be used as tools to investigate gunshot wounds and the lead derived from GSRs in animals. Specifically, the low sensibility of BTK suggests the potential for its use as a screening tool for the rapid and cost-effective detection of lead in close contact shots. Furthermore, the higher sensitivity of NaR and NaR HCL (5%) suggests its validity as a tool to investigate both close contact and intermediate or long-range fire, depending on the firearm used. 

## 6. Limitations

A few limitations of the study should be noted. First, the small sample size and the use of few anatomical regions (limbs) for the experimental part of the study. Second, a limited number of ammunitions were tested. Therefore, further studies will be conducted to better investigate the gunshot wound morphology and lead deposition in relation to different anatomical sites and types of ammunition. 

## Figures and Tables

**Table 1:** Manner of death of the animals died from gun trauma (group A).

Species	Manner of Death
n. 2 cattle (Group A)	animals found shot inside a farm and died after 24 h
n. 1 cattle (Group A)	animal found shot to death inside a farm
n. 3 dogs (Group A)	animals found shot to death—no additional information was reported
n. 2 horses (Group A)	animals found shot to death inside a farm

**Table 2:** Samples, firearms, bullets and shooting ranges used for the experimental section of the study (groups B and C).

Limbs	Weapon	Bullet	Shooting Range
n. 3 cases (group B)	Pistol	9 mm copper-plated bullets	close contact
n. 3 cases (group B)	Pistol	9 mm copper-plated bullets	1 m
n. 3 cases (group B)	Pistol	9 mm copper-plated bullets	6 m
n. 3 cases (group B)	Pistol	9 mm full metal jacket bullets	close contact
n. 3 cases (group B)	Pistol	9 mm full metal jacket bullets	1 m
n. 3 cases (group B)	Pistol	9 mm full metal jacket bullets	6 m
n. 3 case (group C)	Rifle	cal. 12 shotgun slug	6 m
n. 3 case (group C)	Rifle	cal. 12 shotgun slug	12 m

**Table 3:** Location of the gunshot wound in group A animals.

Species	Location of the Gunshot Wound
n. 1 cattle	skull region
n. 1 cattle	abdominal region
n. 1 cattle	chest region
n. 2 dogs	chest region
n. 1 dog	skull and neck regions
n. 2 horses	chest region

## Data Availability

All relevant data are listed in the manuscript and in the Supplementary Materials of this article.

## Supplementary Materials

The following supporting information can be downloaded at: https://www.mdpi.com/article/10.3390/ani14192913/s1; Figures S1–S5: close-contact shots; Figures S6–S11: 1-meter shots; Figures S12–S15: 6-meters shots (Group B cases).
