# Supplementary figures and images for: Pathological Changes and Sodium Rhodizonate Test as Tools for Investigating Gunshot Wounds in Veterinary Forensic Pathology
Source: Animals (Basel). 2024 Oct 9;14(19):2913. doi: 10.3390/ani14192913 (PMC11476102; doi:10.3390/ani14192913)

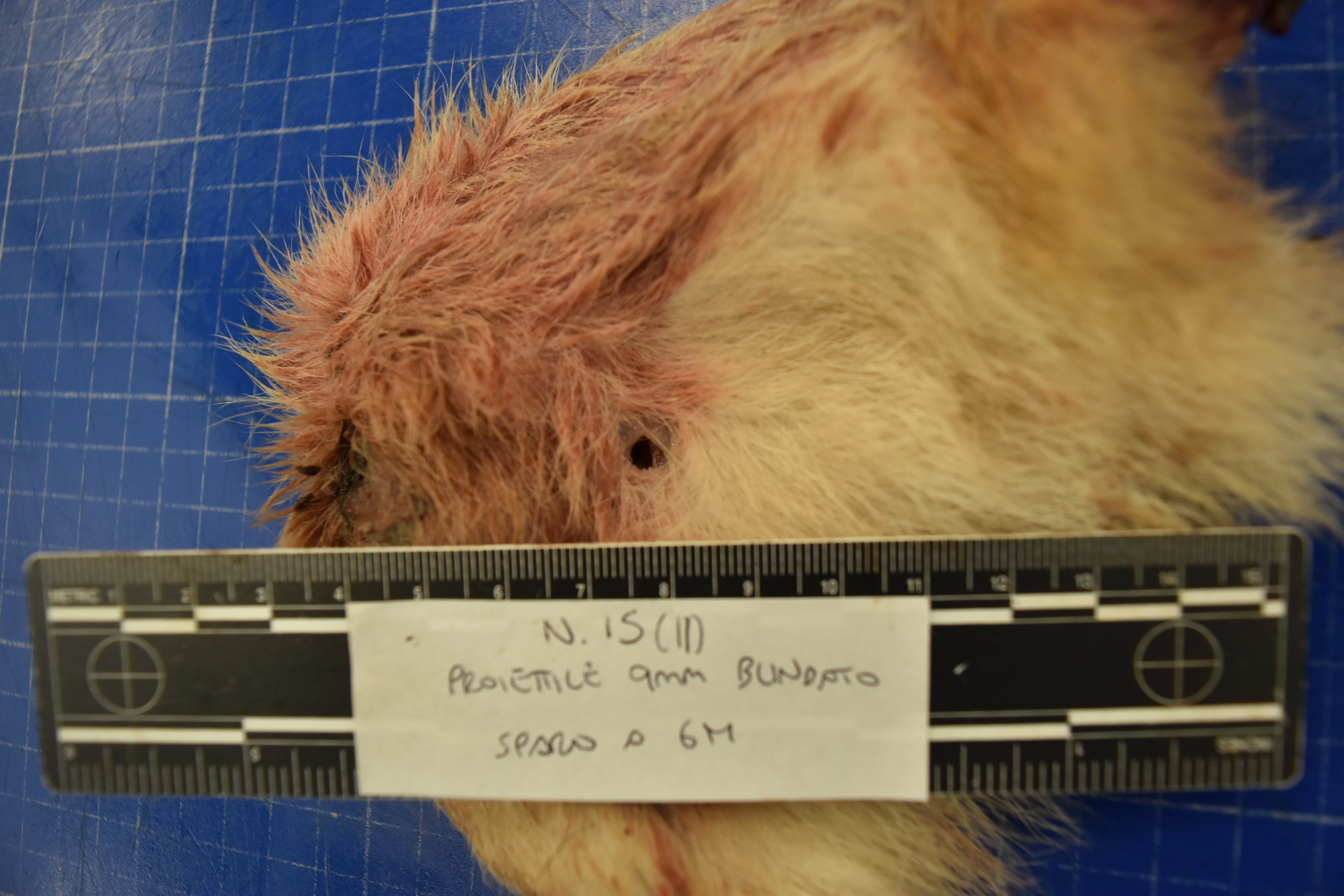

Supplement: Supplementary file 1 [file animals-14-02913-s001.zip › sup. material/fig. 15.JPG]

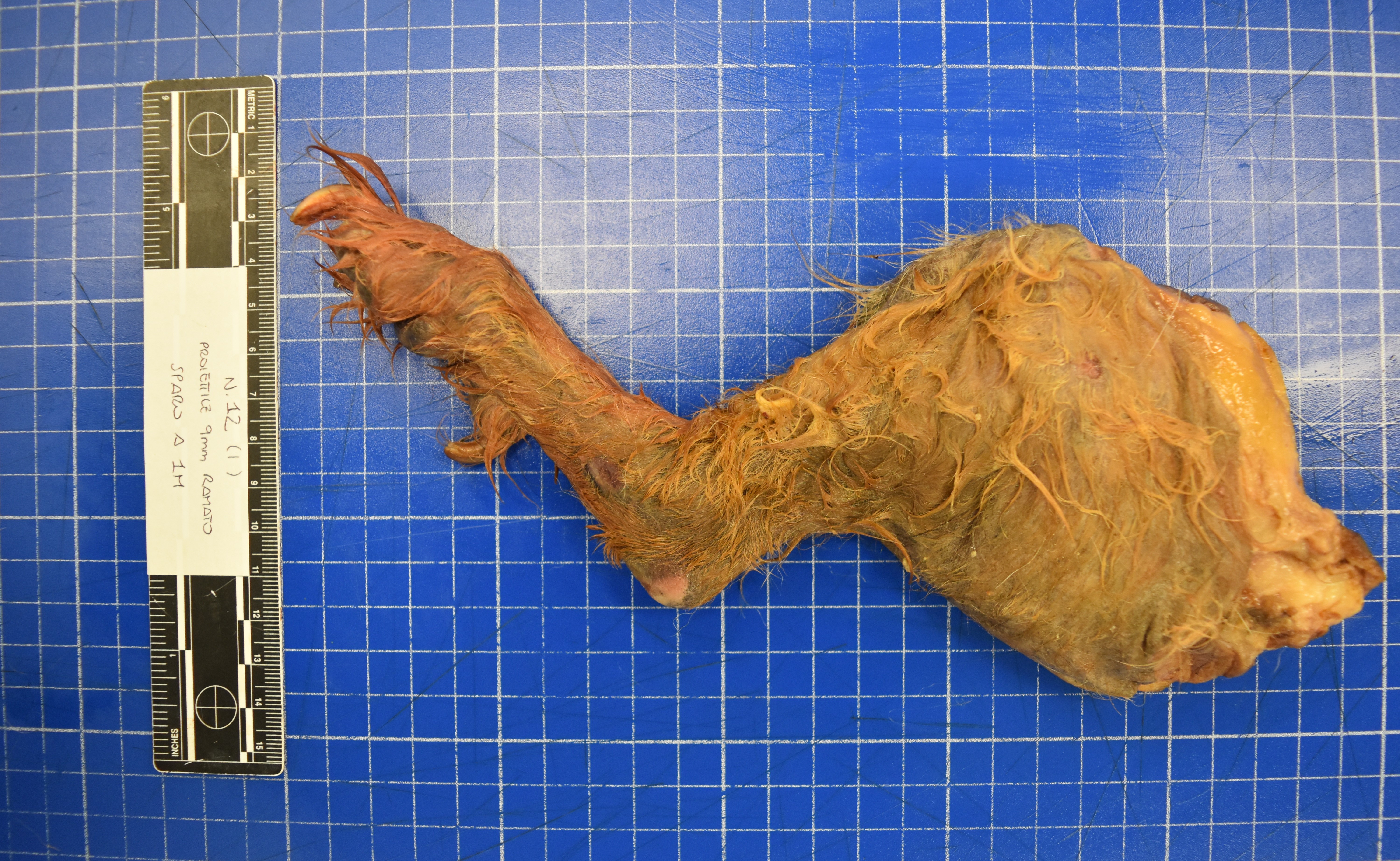

Supplement: Supplementary file 1 [file animals-14-02913-s001.zip › sup. material/fig. 6.JPG]

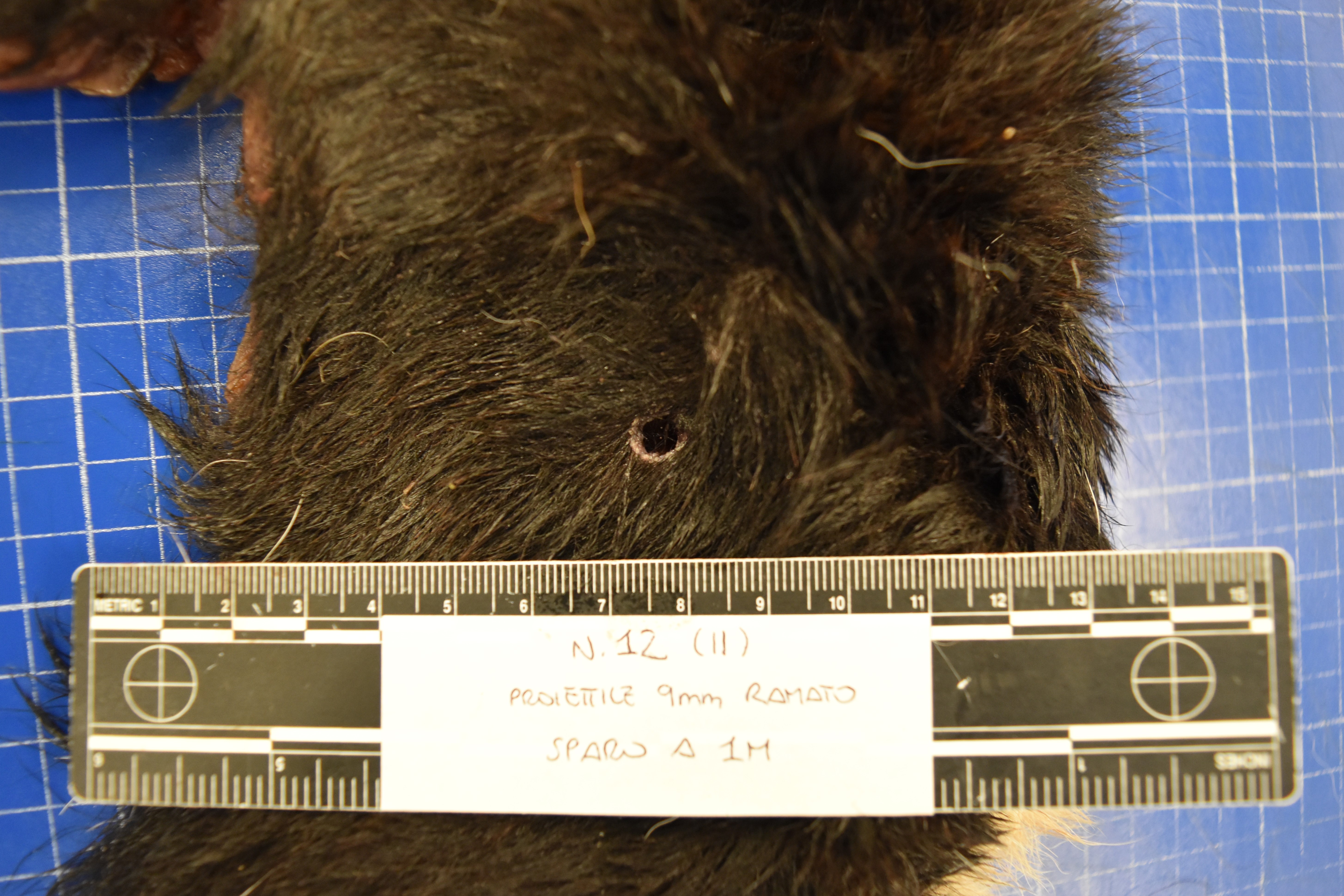

Supplement: Supplementary file 1 [file animals-14-02913-s001.zip › sup. material/fig. 7.JPG]

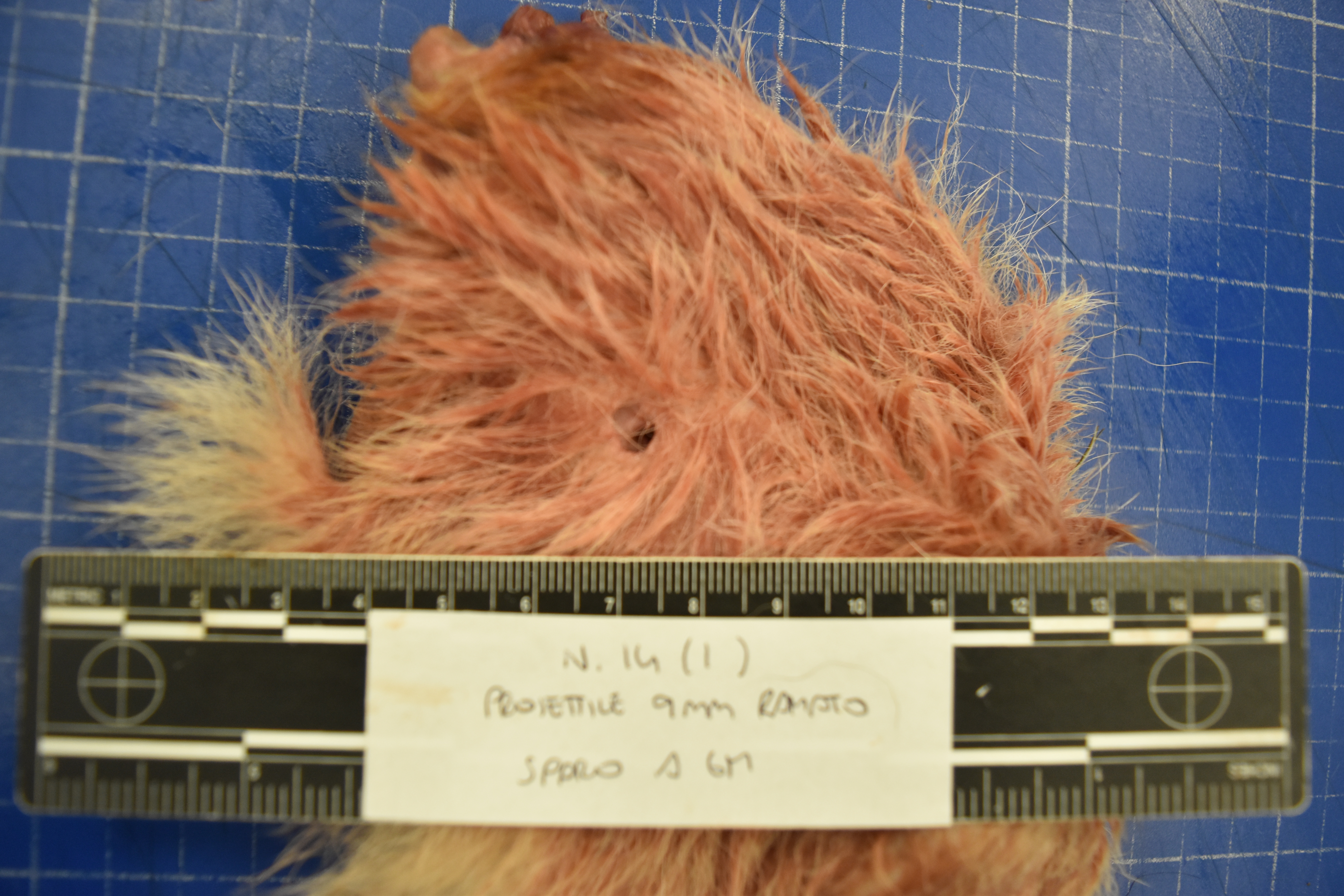

Supplement: Supplementary file 1 [file animals-14-02913-s001.zip › sup. material/fig. 14.JPG]

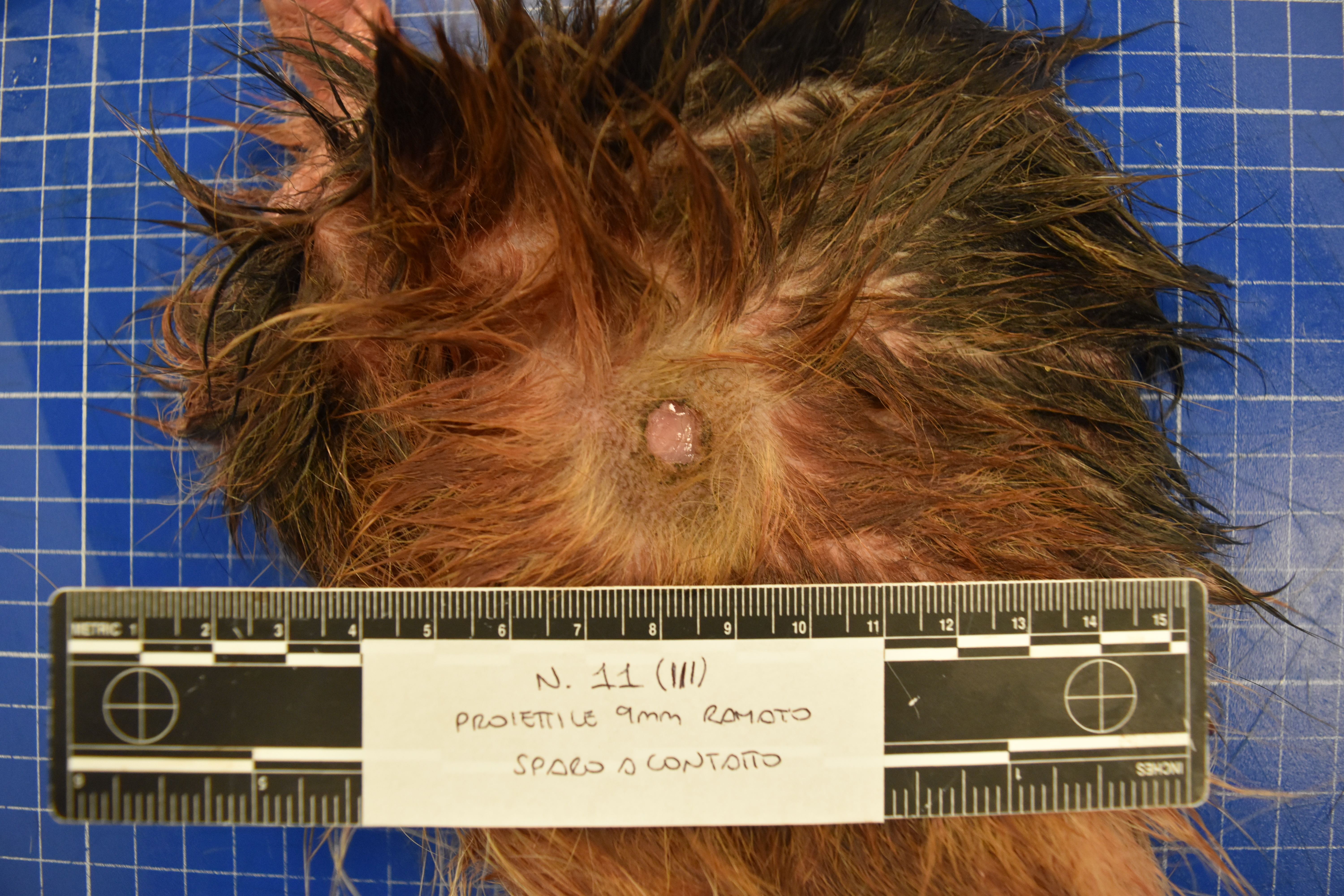

Supplement: Supplementary file 1 [file animals-14-02913-s001.zip › sup. material/fig. 5.JPG]

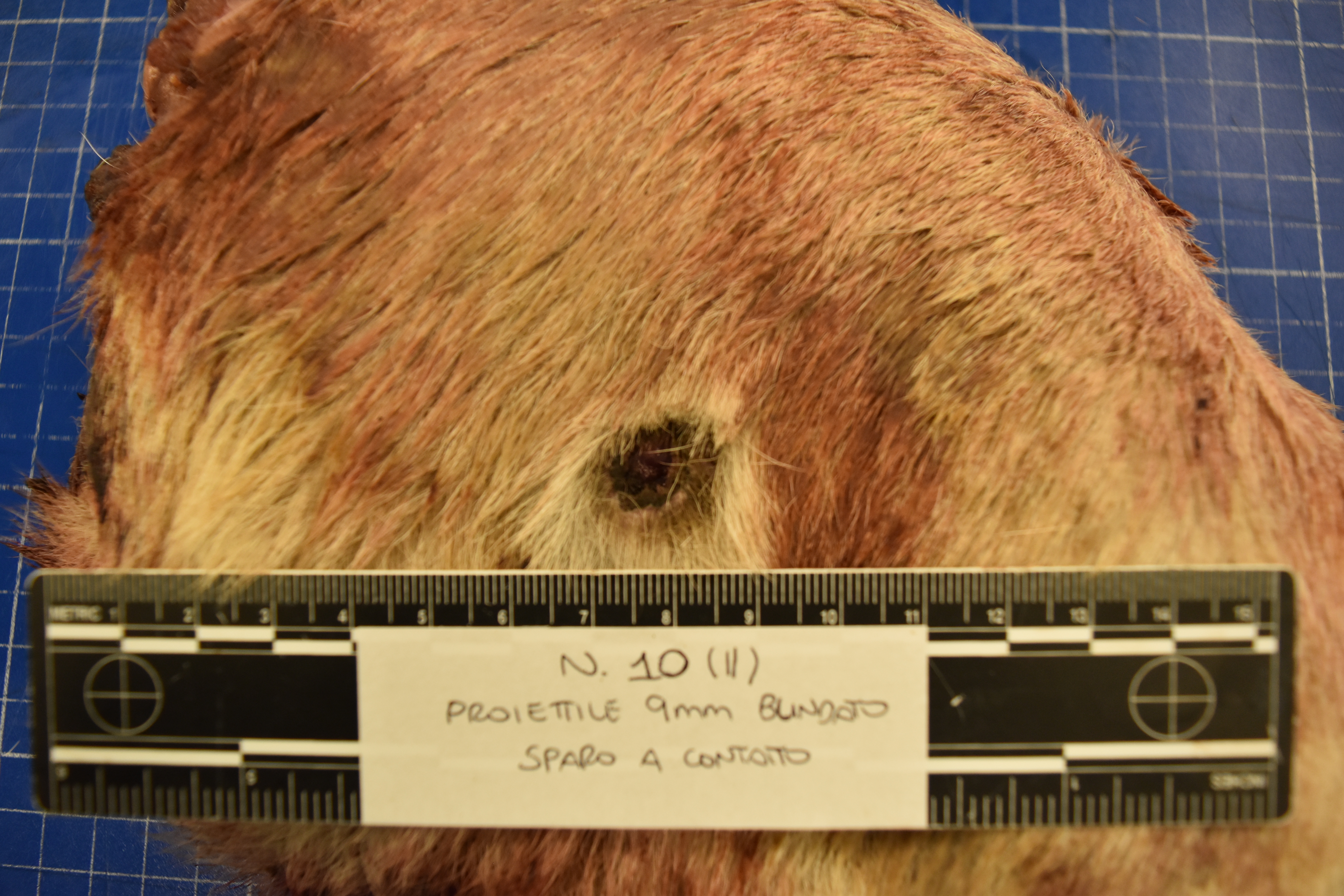

Supplement: Supplementary file 1 [file animals-14-02913-s001.zip › sup. material/fig. 4.JPG]

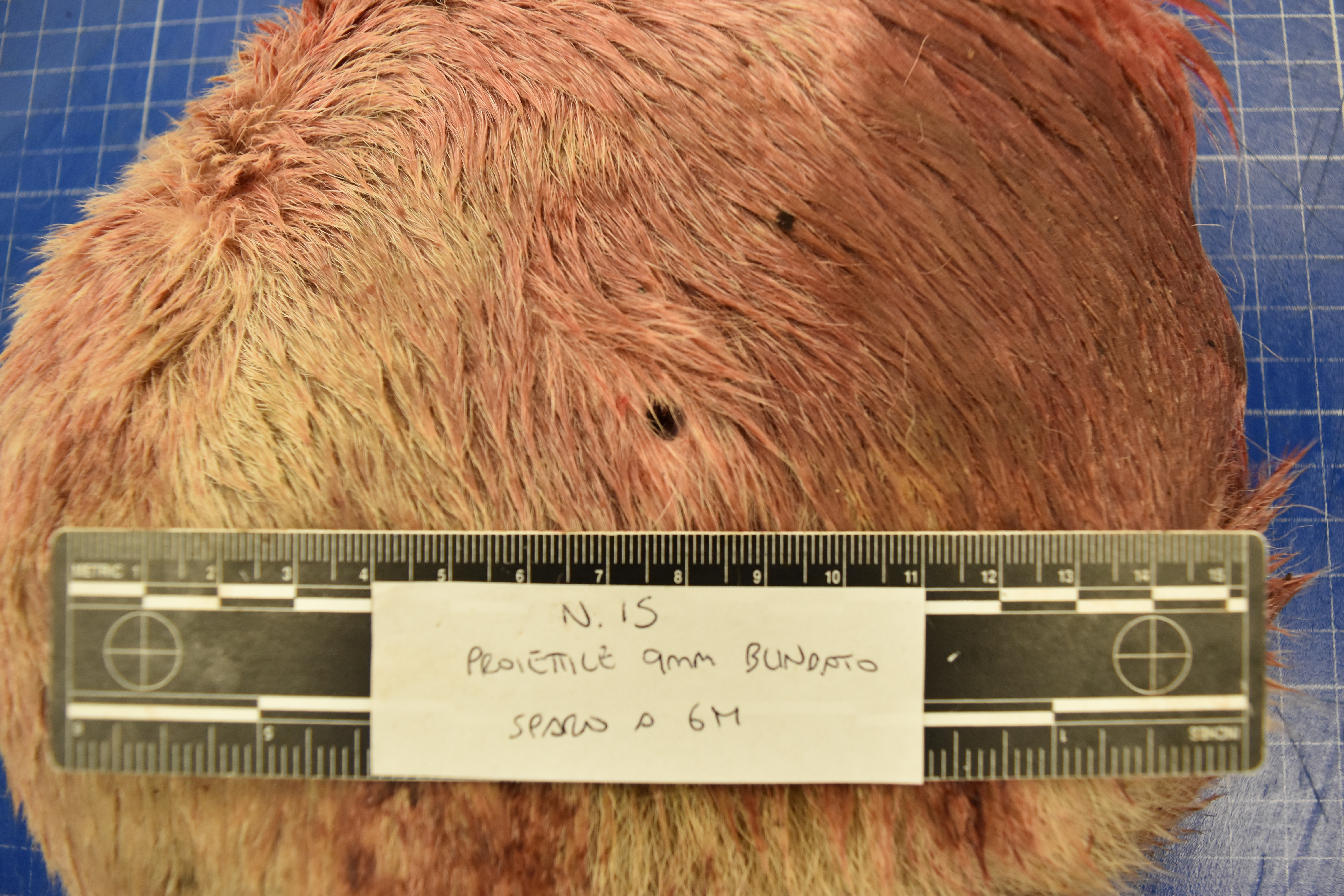

Supplement: Supplementary file 1 [file animals-14-02913-s001.zip › sup. material/fig. 13.JPG]

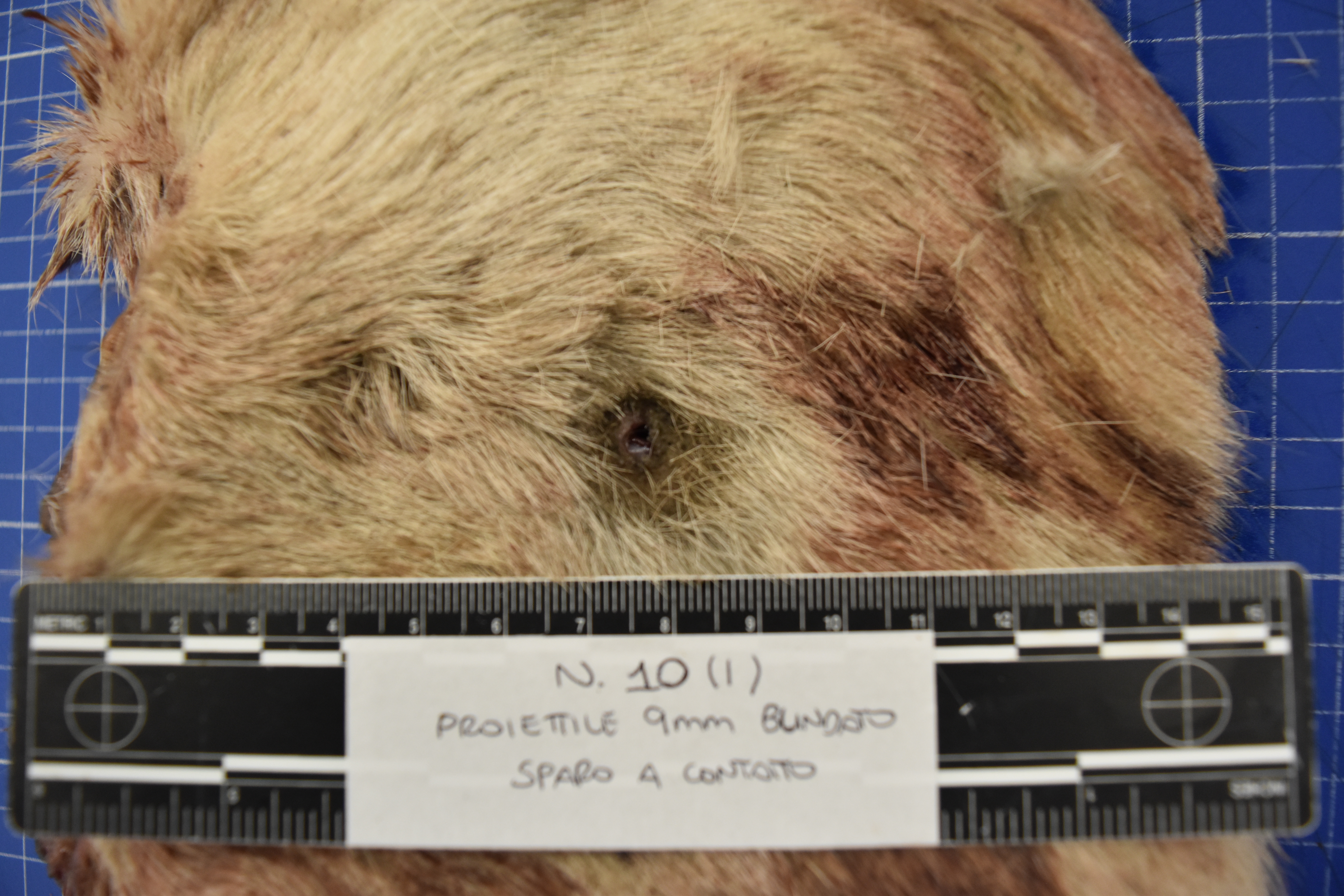

Supplement: Supplementary file 1 [file animals-14-02913-s001.zip › sup. material/fig. 1.JPG]

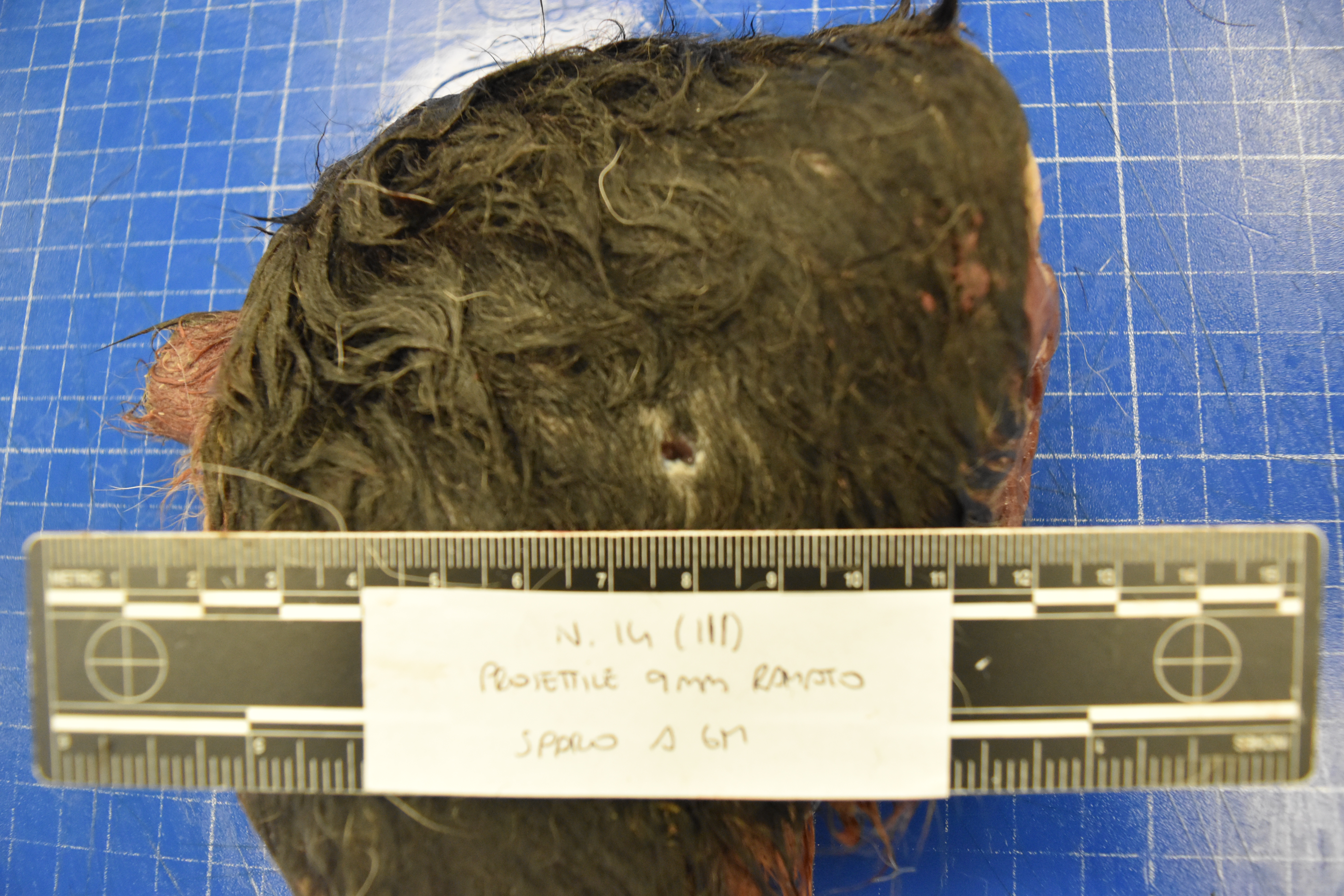

Supplement: Supplementary file 1 [file animals-14-02913-s001.zip › sup. material/fig. 12.JPG]

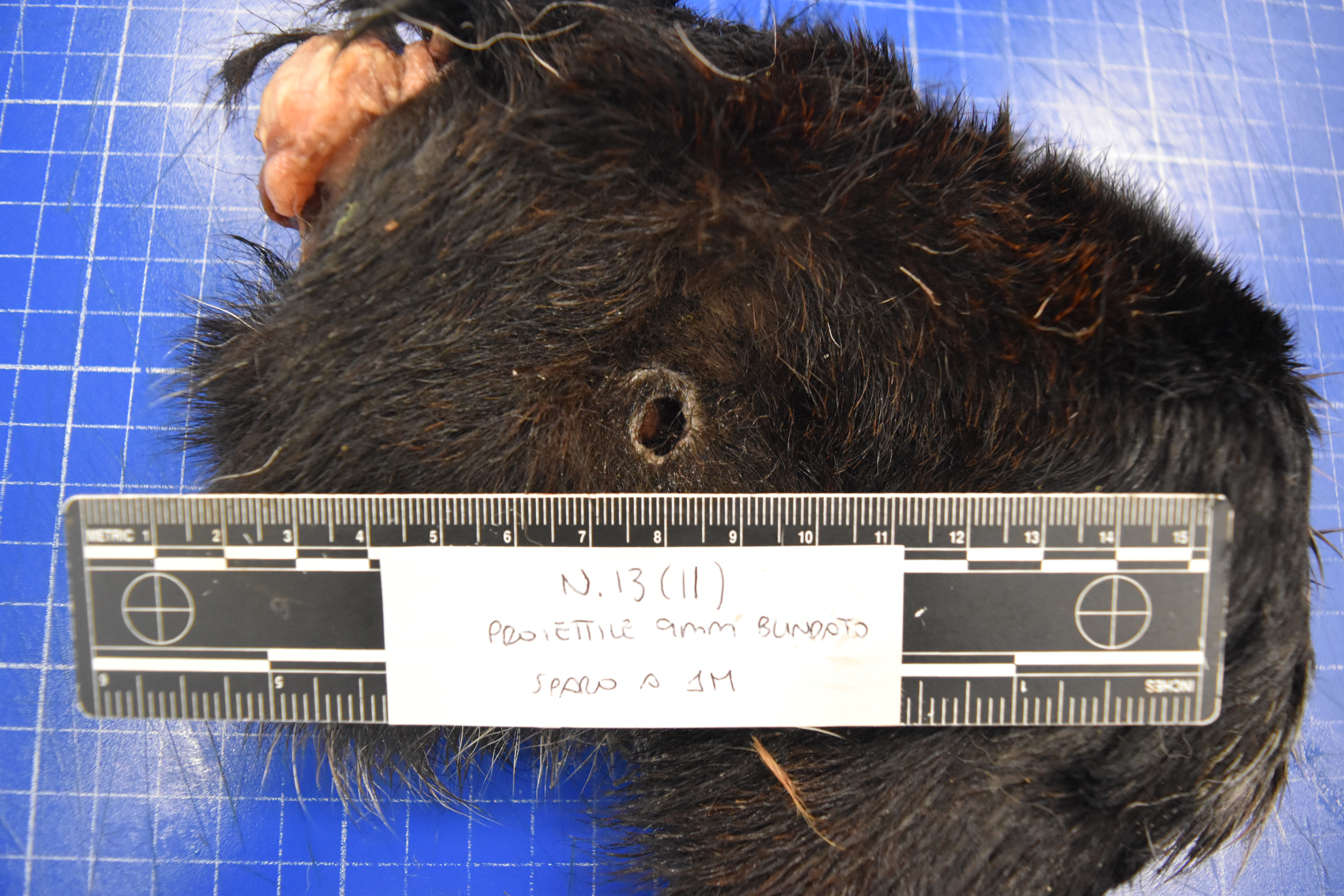

Supplement: Supplementary file 1 [file animals-14-02913-s001.zip › sup. material/fig. 10.JPG]

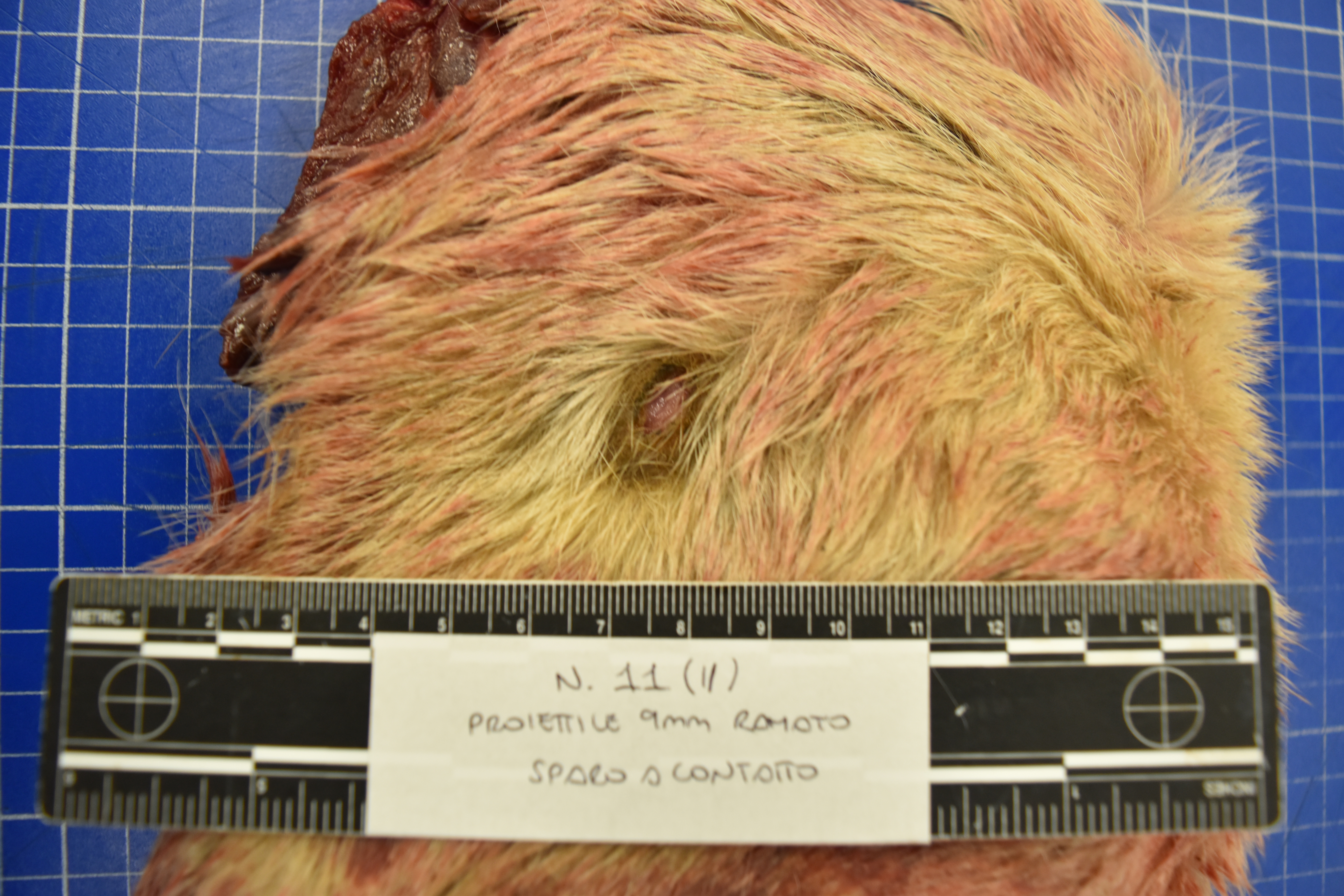

Supplement: Supplementary file 1 [file animals-14-02913-s001.zip › sup. material/fig. 3.JPG]

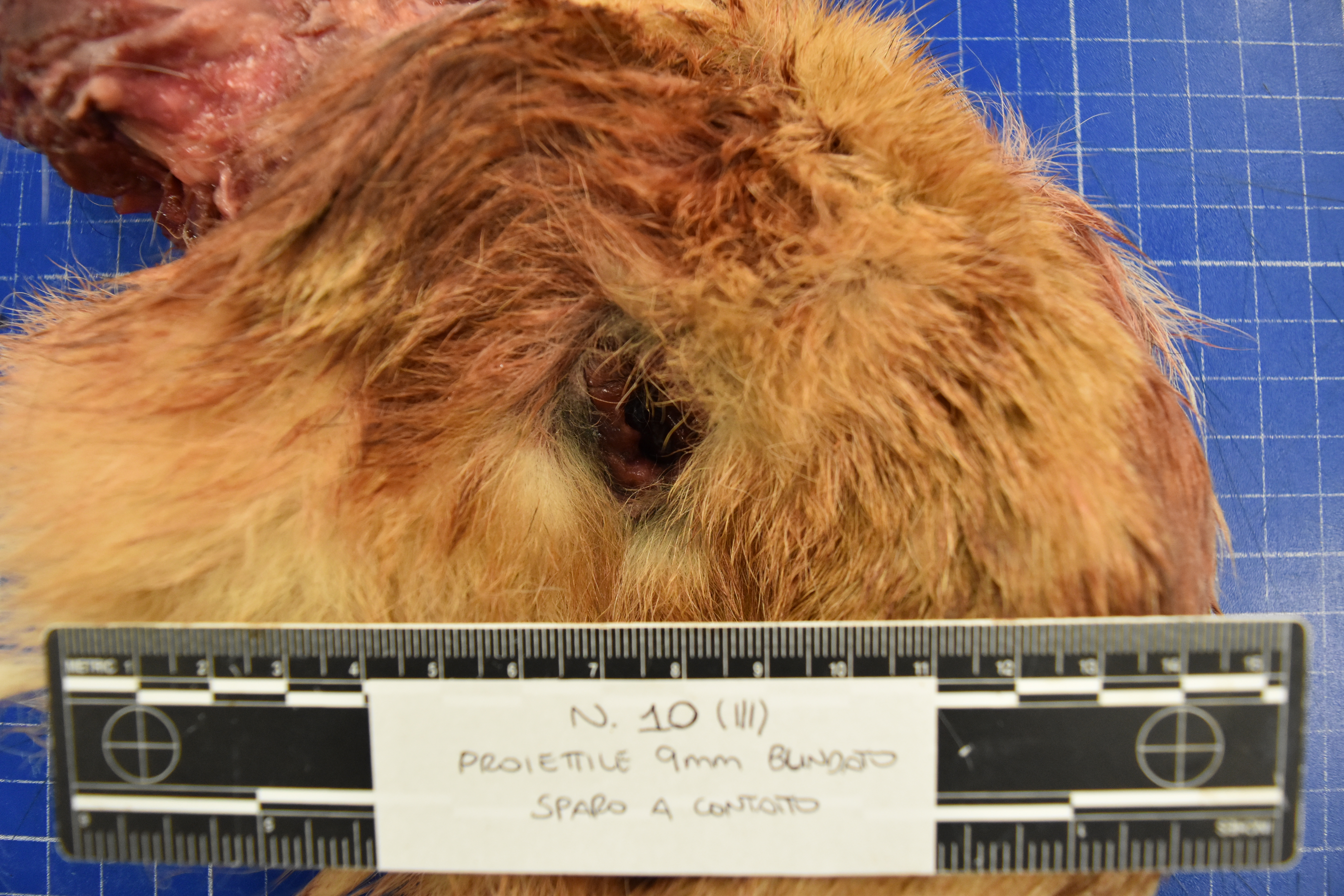

Supplement: Supplementary file 1 [file animals-14-02913-s001.zip › sup. material/fig. 2.JPG]

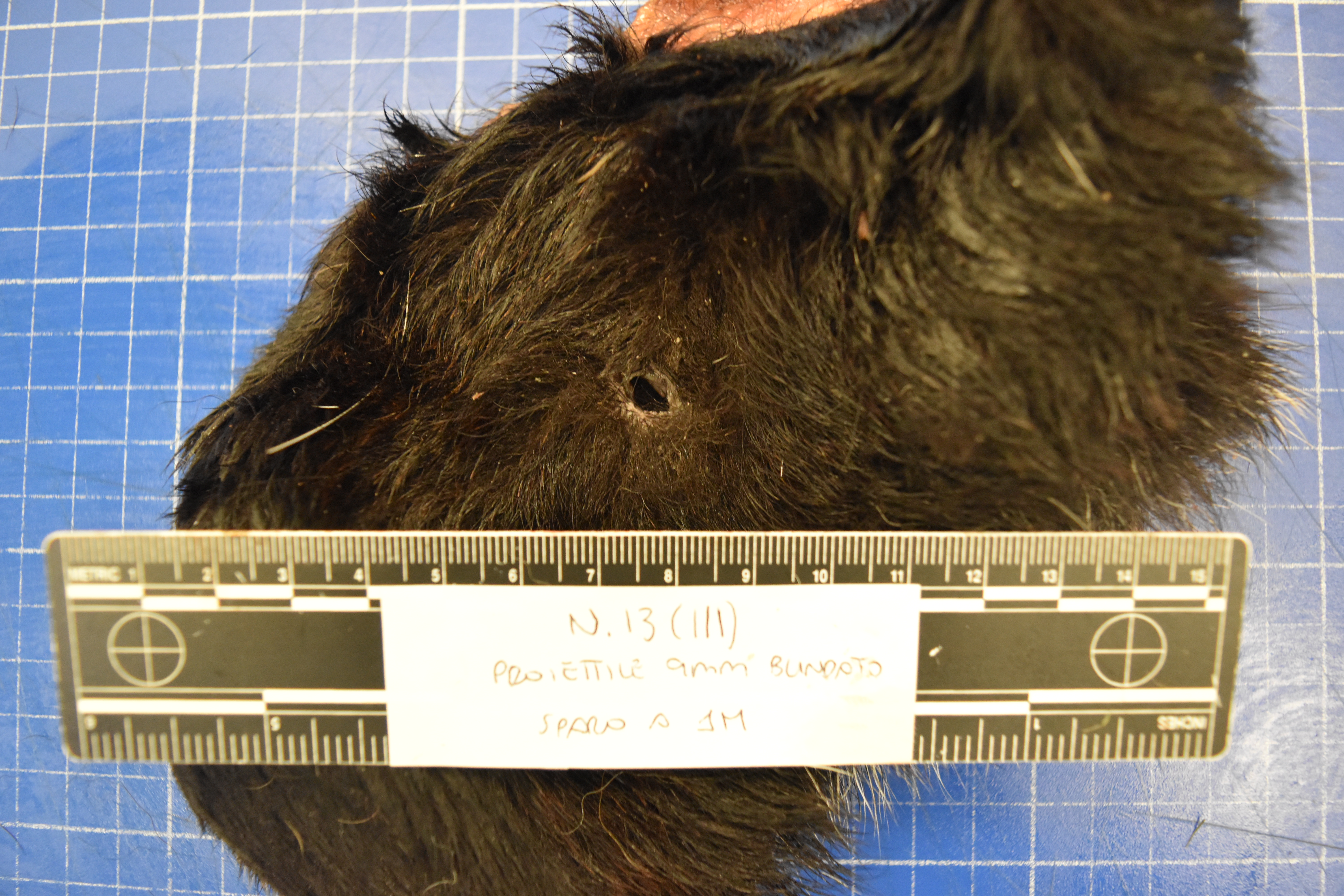

Supplement: Supplementary file 1 [file animals-14-02913-s001.zip › sup. material/fig. 11.JPG]

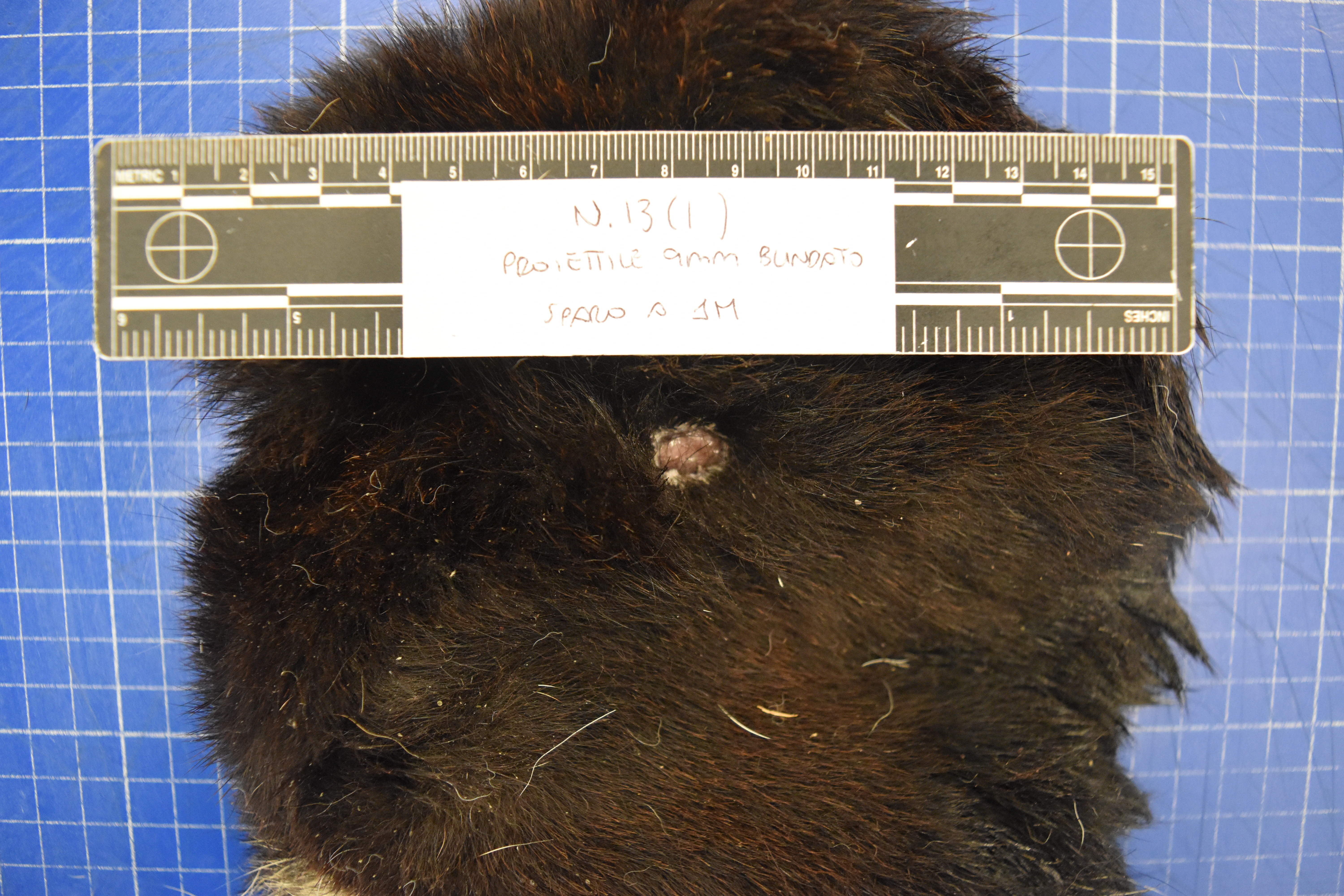

Supplement: Supplementary file 1 [file animals-14-02913-s001.zip › sup. material/fig. 9.JPG]

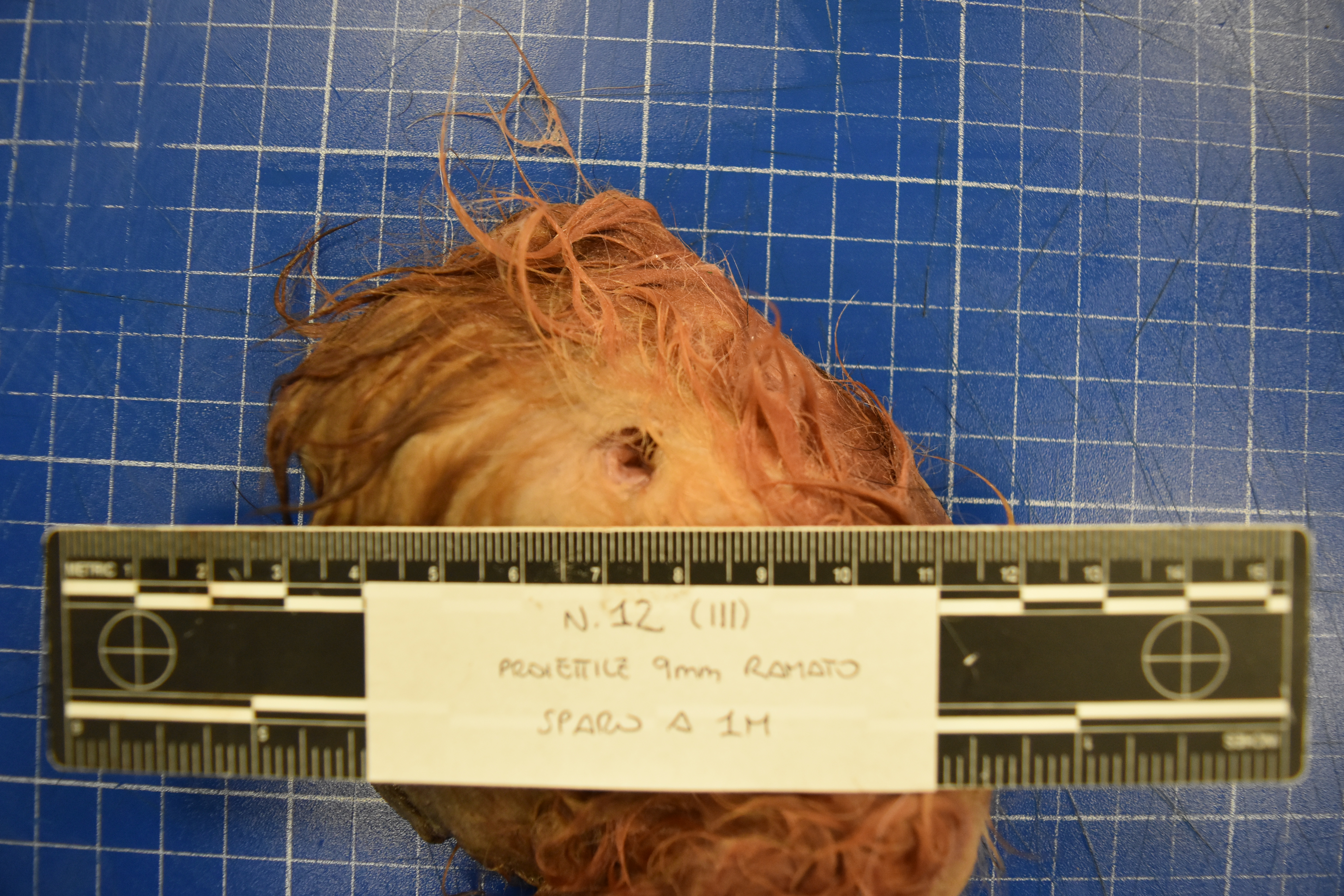

Supplement: Supplementary file 1 [file animals-14-02913-s001.zip › sup. material/fig. 8.JPG]
